# The protective effect of Indian Catechu methanolic extract against aluminum chloride-induced neurotoxicity, A rodent model of Alzheimer's disease

**DOI:** 10.1016/j.heliyon.2021.e06269

**Published:** 2021-02-14

**Authors:** Ekramy Elmorsy, Eman Elsharkawy, Fahad A. Alhumaydhi, Mohamed Salama

**Affiliations:** a Forensic Medicine and Clinical Toxicology Department, Faculty of Medicine, Mansoura University, Egypt; b Pathology Department, Faculty of Medicine, Northern Border University-ARAR, North Region, Saudi Arabia; c Department of Eco Physiology, Ecology and Range Management Division, Desert Research Center, Mathef El-Mataria, 15753 Egypt; d Department of Chemistry, Science Faculty for Girls, Northern Border University-ARAR, North Region, Saudi Arabia; e Department of Medical Laboratories, College of Applied Medical Sciences, Qassim University, Buraydah 52571, Saudi Arabia; f Institute of Global Health and Human Ecology, The American University in Cairo (AUC), Cairo 11385, Egypt

**Keywords:** Alzheimer disease, Indian Catechu, Acetyl cholinesterase and neuro-monamines, Beta-amyloid proteins, Tauopathies, Oxidative stress

## Abstract

Alzheimer's disease (AD) is the commonest neurodegenerative disorder with a wide array of manifestations, courses, and contributing causes. Despite being clinically characterized a long time ago; no treatment has been developed that could improve the pathology or slow down the disease manifestation- so far. Indian Catechu methanolic extract (ICME) has proved to have multiple beneficial effects that support its use in several disorders- especially those with complex etiology. In the present study, we evaluated the neuroprotective effect of ICME in a rat model of AD using Aluminum Chloride (AlCl3). The results showed that ICME could have a positive impact on the course of AD through its anticholinesterase effect and significant antioxidant effect which was reflected on the animals both on behavioral tests as well as hallmark pathological findings.

## 1 Introduction

AD is the commonest cause of dementia that affects more than 35 million people worldwide and this number is believed to reach 65.7 million by 2030. Dementia and cognitive decline induced by AD may increase the accident vulnerability of patients, ranking AD as the fourth commonest cause of death in the elderly population. The number of people with AD is expected to increase substantially in the coming years as the proportion of the population aged 65 years or more rises sharply (Alzheimer's Association, 2019).

The pathogenesis of AD is not identified, however, AD neuropathology is generally characterized by neuritic plaques, neurofibrillary tangles, and cholinergic neurons loss in the nucleus basalis of Meynert. Multiple etiological factors have been suggested to contribute to its pathogenesis. Some risk genes have been suggested to increase the deposition of beta-amyloid plaques, as well as abnormal phosphorylation of tau protein to form the common neurofibrillary tangles (Kunkle et al., 2019). Also, inflammation, oxidative stress (Simunkova et al., 2019), hormonal deficiency (estrogen) (Kong et al., 2019), and aging altogether have a corroborative role (Selkoe, 2019). Also, according to the cholinergic theory, the development of Alzheimer's disease symptoms is mainly related to structural alterations in cholinergic synapses, loss of specific subtypes of acetylcholine (ACh) receptors, the death of ACh-generating neurons, and, consequently, the deterioration of cholinergic neurotransmission. These issues lead to a relative accumulation of the ACh-hydrolyzing enzyme, acetylcholinesterase (AChE) (Stanciu et al., 2020).

Aluminum is the third most common element in the earth's crust with high levels of the human being expected exposure through cooking utensils, food antacids, and deodorants as well as various industrial applications (Singh and Goel, 2015). Aluminum is known as a potent neurotoxic agent that can pass through the blood brain barrier under normal conditions and is widely distributed to different brain areas. Aluminum has been shown to induce brain oxidative damage, neuronal death, cholinergic neurons degradation, and amyloid deposition with subsequent memory and learning deficits (Alexander et al., 1996; Germano and Kinsella, 2005; Miu et al., 2006). Also, Aluminum is considered as a potentially hazardous environmental agent with a suspected role in the development of Alzheimer disease (AD) as higher levels of aluminum has been reported in AD patient brains with noticed higher levels in the hippocampus (Rondeau et al., 2009; Kaur and Gill, 2006). Additionally, Aluminum causes misfolding to the cytoskeleton proteins which leads to the formation of amyloid plaques and the neurofibrillary tangles in the brain (Campbell et al., 2000; Kawahara et al., 2001). Hence, it is widely accepted to use Al for inducing neurodegenerative changes in animals to simulate AD.

Currently, the available drugs for AD are predominantly cholinesterase inhibitors. However, the efficacy of these drugs is limited as they are not able to completely arrest the progression of the disease (Sharma, 2019). This could be attributable to the complex nature of the disease etiology. As such, a drug that comprises different protective functions alongside anticholinesterase effects, could offer a promising therapeutic approach for AD.

Numerous plants have been reported to have a therapeutic benefit in cases with impaired memory, Alzheimer's disease (AD), and old age-related diseases. Interestingly, in the last 20 years, some medicinal plant preparations have proved effective in slowing down dementia and AD manifestations (Tian et al., 2010).

Acacia catechu, also known as kattha (Urdu), is a common plant in India, other Asian countries, and East Africa. Traditionally, A. Acacia catechu (*A. catechu*), commonly known as “khair” in India has well-known reported medicinal uses (Hazra et al., 2010) due to the variety of actions it has e.g. anti-inflammatory, antidiabetic, antioxidant and anticholinesterase effects (Ray et al., 2006; Wadood et al., 2007). The potential to use the Indian Catechu methanolic extract (ICME) in the management of neuro-degenerative diseases has not been investigated. Hence, the current study was conducted to evaluate the anti-Alzheimer's effects of Indian Catechu methanolic extract on an animal model using aluminum chloride-induced neurodegeneration.

## 2 Materials & methods

### 2.1 Ethical issues

This study was conducted in accordance to the ethical procedures and policies of the local bioethics committee of Northern Border University, Saudi Arabia. Forty male Wistar rats weighing approximately 250 ± 50 g were used in this study. The rats were accommodated in well-ventilated polypropylene cages with maintained 12/12 h' light dark-cycle at a temperature of 25 ± 3 °C and 30%–60% humidity with free access to standard food and water. Rats were allowed to acclimatize to these conditions for one week before the experiments. Then the experiments were conducted between 09.00 and 17.00 h in the light phase.

### 2.2 Chemicals

All chemicals used in the study were purchased from Sigma Aldrich (St Louis, MO, USA) unless another source is specified.

### 2.3 Indian Catechu methanolic extract (ICME) preparation

Commercial *catechu* was purchased from the local market as it is widely sold as a food additive. It is prepared by boiling with the evaporation of the resulting brew of A. Catechu bark. The presence of *Uncaria gambier* was excluded by negative gambier fluorescein test results (Ray et al., 2006). 75 gm of the powder was exhaustively extracted with 95% methanol. The methanol extracts were filtered, evaporated under vacuum at 35 °C, freeze-dried, and stored at -20 °C (Patil and Modak, 2017). Further dilutions of the extract were prepared in water.

### 2.4 Anti-cholinesterase (AChE) activity assay

AChE activity was measured based on the method of Ellman et al. (1961), adapted for a microtitre plate format following Nwidu et al. (2017). The anticholinesterase activities of the ICME were tested in concentrations 0.2, 2, 20, and 200 μg/mL. A negative control assay performed in the absence of AChE provided a reagent blank. Eserine (3 μM) was used as a well-known positive control. The percentage inhibition of AChE by ICME was calculated relative to inhibition by eserine, assuming that eserine (3 μM) produces 100% inhibition of the enzyme activities. Experiments were conducted in triplicates for the different studies concentrations.

### 2.5 2,2-Diphenyl-1-Picrylhydrazyl (DPPH) radical scavenging assay

The antioxidant activities of the extract were assessed by DPPH radical scavenging assay following Nwidu et al. (2017) using final concentrations of 1, 10, 100, and 1000 μg/mL. Ascorbic acid was used as a positive control. The radical scavenging capacity of extract was measured by calculating the percentage of DPPH radical scavenged. Experiments were conducted in triplicates for the different studies concentrations.

### 2.6 Aluminum chloride toxicity model induction

Thirty randomly selected rats were divided into four groups containing 10 animals each. Group I: Rats treated with normal saline by oral gavage. Group II: Rats induced with AlCl3 100 mg/kg b. wt. daily for 60 days by oral gavage (Nampoothiri et al., 2015). Group III: Rats induced with AlCl3 as group II and subsequently received *ICME* (3 mg/kg/day) I.P. on daily basis for 15 days. After 24 h of the administration of the last dose, Blood samples were collected in ethylene diamine tetraacetic acid (EDTA) anticoagulant tubes. Then the rats were sacrificed by decapitation under thiopental anesthesia.

### 2.7 Neurobehavioral studies

#### 2.7.1 Open field

Locomotor and behavioral activities were assessed by the open field activity monitoring test following Rajasankar et al. (2009) using a wooden apparatus that is divided into 16 (4 × 4) squares. The rat is placed inside the box and given the time to familiarize with the test atmosphere then it was observed for 5 min for the number and pattern of explored squares, the total time of immobility (measured in seconds), rearing number, and the number of fecal pellets.

#### 2.7.2 Morris water maze

The effect of AlCl3 and ICME on the retention of working and spatial memory in rats was assessed by Morris water maze (Morris, 1984; Nunez, 2008). The animal was allowed for 120 s to locate the platform and if it failed it is guided to it. While the animal was allowed to stay on the platform for no more than 20 s. Spatial memory was recorded by the time taken (S) by the rat from its immersion point till reaching the platform from immersion into the pool. While working memory was recorded by the time spent (S) by the rat in the target quadrant (maximum of 120 s) without the platform.

#### 2.7.3 Novel object recognition

Novel object recognition was performed according to Antunes and Biala (2012) method. During the retrieval phase, the animal was allowed to explore the field for 10 min. The hippocampal functions were assessed through the discrimination and recognition indices, which were measured according to the time spent by the animals to explore the familiar and new objects.

### 2.8 Tissue collection

After decapitation, the whole brain of each animal was rapidly dissected, thoroughly washed with isotonic saline, dried, weighed, and then divided mid-sagitally into two halves. The left hemisphere was specified for histopathological studies which the right half of each brain was homogenized immediately following Carter et al. (2007). The protein content of the brain homogenate was estimated by Lowry method (Lowry et al., 1951) for further biochemical investigations. For DNA fragmentation assay, part of the hemisphere was lysed were homogenized in a lysis buffer l (pH 8.0) using 10 times their volume following Wu et al. (2002).

### 2.9 Acetylcholinesterase assay

The AChE activity was measured using Ellman method (Ellman et al., 1961) as shown before using the brain homogenates of the control and treated animals as a source of cholinesterase enzyme. The brain homogenate was prepared from the right cerebral hemisphere cortical and hippocampal areas following Carter et al. (2007).

### 2.10 Biogenic amines assay

Reversed-phase High Performance Liquid Chromatography (HPLC) with an electrochemical detector was performed to detect levels of biogenic amines [Noradrenaline (NA), dopamine (DA), 5-hydroxytryptamine (5-HT)] in brain homogenates samples (Haider et al., 2011, 2014).

### 2.11 Biochemical markers of oxidative stress

#### 2.11.1 Reactive species production

Reactive Oxygen Species and nitrogen-oxygen species levels were investigated as biomarkers of oxidative stress. Reactive oxygen species (ROS) in the collected brain tissues were measured following Singh et al. (2018). The level of ROS was measure by spectrophotometery using 499 nm and 520 nm wavelengths as excitation and emission wavelengths respectively.

#### 2.11.2 Lipid peroxidation (LPO)assay

LPO was estimated by thiobarbituric acid reactive substances (TBARS) according to the method of Niehaus and Samuelsson (1968) using tertiary butanol−trichloroacetic acid−hydrochloric acid (TBA–TCA–HCl) reagent. The fluorescence of light pink colored supernatant was read at 532 nm. LPO data was represented as μmol of MDA/g of the brain.

#### 2.11.3 Reduced glutathione

Reduced glutathione (GSH) content in the brain samples was studied according to Ellman (1959) protocol. The activity of GSH was expressed as nM GSH/100 g tissue.

#### 2.11.4 Antioxidant enzymes activities

Catalase (CAT) activity was assessed according to Sinha (1972) protocol using a colorimetric assay. Briefly, tissue homogenate (0.1 ml) was added to 1 ml of 0.01 M phosphate buffer (pH = 7) and 0.4 ml H_2_O_2_ (2M). Finally, 2 ml of dichromate-acetic acid was added to stop the reaction and the absorbance was read at 620 nm and presented as micromoles of H_2_O_2_ consumed per minute per mg of protein.

The assay for superoxide dismutase (SOD) was performed using a commercial kit ab65354 (Abcam) following the manufacturer protocol. The principle of the assay was based on the ability of SOD to inhibit the reduction of nitroblue tetrazolium to blue formazan by superoxide anions. Tissues were homogenized and centrifuged at 14000 for 5 min at 4 °C. The supernatant, containing total cytosolic and mitochondrial SOD enzymes, was collected. Twenty μl of each sample was added to the reaction mix in the 96 well-plate and incubated for 20 min in 37 °C. Then the absorbance was read at 450 nm. SOD activity was normalized to the total tissue proteins of the tested samples in mg. A standard curve was prepared using SOD obtained from Abcam.

### 2.12 DNA fragmentation assay

The amount of fragmented DNA was determined as described previously by Wu et al. (2002) with slight modifications. Homogenized Brain tissues were centrifuged at 27,000 ×g for 20 min to separate the intact DNA in the cell pellet from the fragmented portion of the supernatant. Both the pellet and the supernatant were suspended in 1 ml of TE buffer (pH 8.0) containing 10 mM Tris-HCl and 1 mM EDTA and treated with diphenylamine reagent. The absorbance to obtain a blue-colored complex was read at 595 nm.

### 2.13 Genes expression assay (rtPCR)

Trizol reagent (Life Technologies, Grand Island, NY) was used to extract the total RNA of the brain samples following the manufacturer's protocol. The extracted RNA quantity and purity were measured by NanoDrop 2000c (Thermo Fisher Scientific, Waltham, MA). The targeted genes and their primers used in the experiment were shown in Table 1. β-actin gene expression was used as an internal standard to normalize the targeted gene expression. cDNAs strands were synthesized using a Reverse Transcription System Kit (Promega, Madison, WI). The qRT-PCR was performed using HotStart-IT® FideliTaq™ PCR Master Mix (2X) (catalog no. 71156, Affymetrix, USA). The thermocycling conditions were adjusted to 95 °C for 10 min; 40 cycles at 95 °C for 7 s, 57 °C for 30 s, and extension at 72 °C for 30 s. Reactions were performed using the CFX96 real-time System (Bio-Rad Laboratories, Inc.). genes expressions were calculated and normalized to the internal reference gene β-actin expression. Then values of controls were normalized to one for easier data expression. Experiments were done in triplicates.

**Table 1:** The gens evaluated by rt-PCR and their forward and reverse primers used in the study.

Genes	Forward primers	Reverse primers
CAT	5′GTGAGAACATTGCCAACCAC3′	5′CTCGGGAAATGTCATCAAAAG3
Sod1	5′CACTGCAGGACCTCATTTTAATCC3′	5′GTCTCCAACATGCCTCTCTTCAT3′
SOD2	5′CGCTGGCCAAGGGAGAT3′	5′CCCCGCCATTGAACTTCA3′
Cas-3	5′AGGCCGACTTCCTGTATGCTT3′	5′TGACCCGTCCCTTGAATTTC3′
Bax	5′CTCAAGGCCCTGTGCACTAAA3′	5′CCCGGAGGAAGTCCAGTGT3′
Bcl-2	5′GGAGGCTGGGATGCCTTTG3′	5′CTGAGCAGCGTCTTCAGAGACA3′
β-actin	5′CCTCTATGCCAACACAGTGC3′	5′AAGCCATGCCAAATGTCTC3′

### 2.14 Western blot

Cortex and hippocampus homogenate proteins were extracted and quantified following Lowry method. Western blotting for proteins Amyloid β1-42, phosphorylated Tau, Caspase 3, Bax, Bcl2, CAT, SOD1, SOD2 and the housekeeping protein β-actin, as a well-recognized internal control, following Singh et al. (2018). The following primary antibodies were used; anti-Aβ1-42 and anti p-Tau (Beijing Biosynthesis Biotechnology Co., Ltd.), mouse monoclonal caspase-3 p17, (1:1000; Santa Cruz Biotechnology), anti-Bax (1:400, Cell Signaling Technology, Danvers, MA, USA), anti-Bcl-2 (1:400, Santa Cruz Biotechnology, Santa Cruz, CA, USA), anti-actin (1:50,000; Millipore). Secondary antibodies goat anti-rabbit secondary antibody, and horseradish peroxidase-conjugated anti-mouse antibody (1:20,000) were used accordingly (Both Santa Cruz Biotechnology). Then the membranes were washed thrice for 15 min in TBST. The protein bands were visualized by Chemiluminescence-based technique using Western blotting substrate kit (Thermo Scientific). Finally, proteins bands density was evaluated using GelQuant.NET software provided by biochemlabsolutions.com. The estimated density was normalized in relation to the housekeeping total actin bands’ density before their statistical analysis.

### 2.15 Histological investigation

The second portions of the sacrificed rats’ brains were used for histopathologic examination, by fixation with10% buffered-saline formalin. Autopsy samples were taken from the brain of rats in different groups and fixed in 10% formol saline for twenty-four hours. Washing was done in tap water then serial dilutions of alcohol (methyl, ethyl, and absolute ethyl alcohol) were used for dehydration. Specimens were cleared in xylene and embedded in paraffin at 56° in a hot air oven for twenty-four hours. Paraffin bees wax tissue blocks were prepared for sectioning at 4 microns' thickness by slide microtome. The obtained tissue sections were collected on glass slides, deparaffinized, stained by hematoxylin & eosin stain for examination through the light electric microscope (Banchroft et al., 1996).

#### 2.15.1 Immunohistochemistry

As beta-amyloid and tau are key factors in etiopathogenesis of AD, immunohistochemistry for Aβ1-42 and phosphorylated (p)-Tau in brain tissue in the rats' brain tissue was conducted following Delcambre et al. (2016). The slides were incubated with antibodies against anti-Aβ1-42 and anti p-Tau (Beijing Biosynthesis Biotechnology Co., Ltd.) After washing with PBS, the slides were incubated with a horseradish peroxidase -conjugated secondary antibody (1:2,000; cat. no. sc-7074; Santa Cruz Biotechnology, Dallas, TX, USA) at 25 °C for 4 h. The slides were visualized with 3,3′-diaminobenzidine and Mayer's hematoxylin at room temperature for 3 min. Then the slides were dehydrated with ethanol and xylene. And images were captured using an Olympus IX73 light microscope (magnification, x100; Olympus, Tokyo, Japan). ImageJ software was used to evaluate the intensity of Aβ1-42 and p-Tau expression.

#### 2.15.2 The TUNEL staining assay

TUNEL staining is mainly used as an apoptosis marker. TUNEL evaluation was typically carried out according to Smith et al. (1997) protocol. Briefly, the brain sections were incubated with 100 mL proteinase K solution (20 mg/ml) then 100 mL of H_2_O_2_ (3%), and washed with PBS two times. After washing, the TUNEL reaction mixed solution was added and the slide was kept in a humid chamber for an hour. Then, 50 mL TUNEL was added and the slide was counterstained with hematoxylin for light microscopy.

### 2.16 Statistical analysis

One-way ANOVA with Tukey posttest was used for statistical analysis of the 3 experimental groups’ data. Significance was considered as p-value<0.05. Graph pad prism 5 software was used for all statistical analyses.

## 3 Results

**Figure 1:**
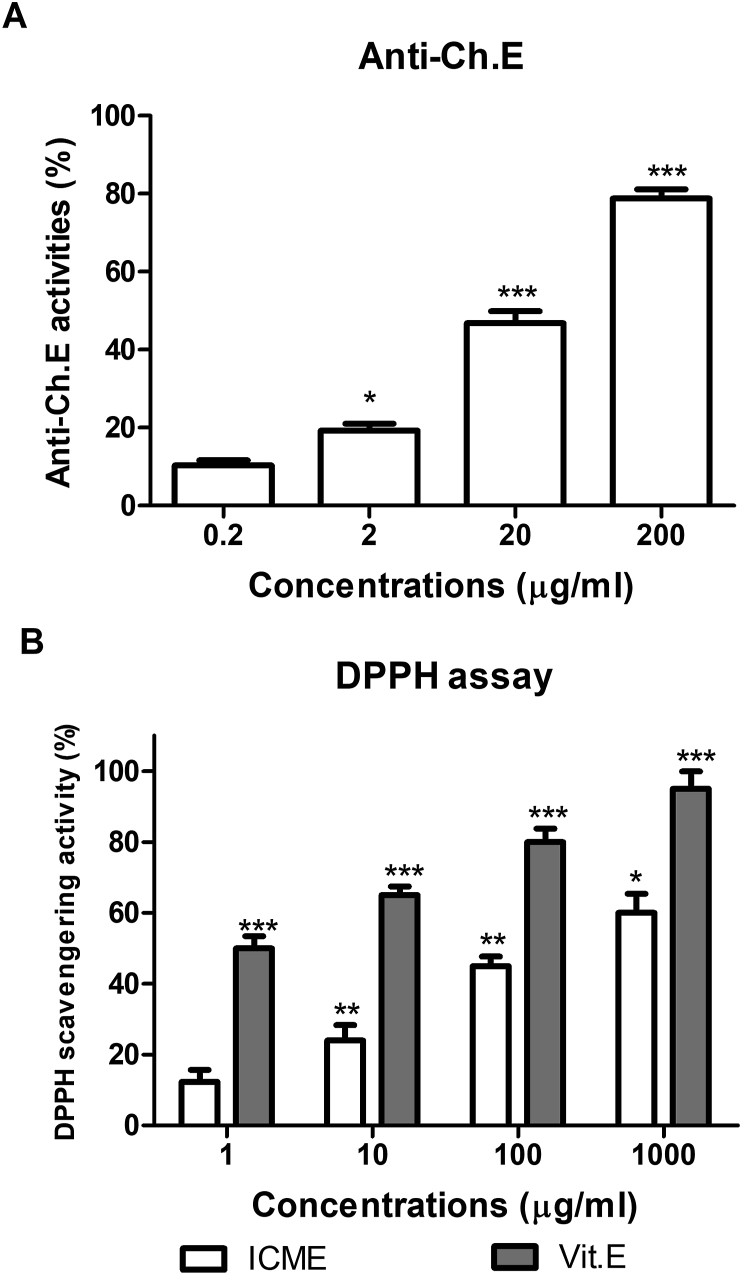
A shows the antichoinestrase activity of Indian Catechu methanolic extract (ICME) according to the modified Ellman assay. Inhibition was shown as a percent of eserine atichoinestrase activity. B shows the DPPH radical scavenging activity of Indian Catechu methanolic extract (ICME). Antioxidant activity of ICME was evaluated as percent of inhibition of DPPH. Vitamin E was used as a positive control. Results were expressed as means ± SD. ∗ means p-value <0.05, ∗∗ means p-value <0.01, while ∗∗∗ means p-value <0.001 relative to control sample (Samples without ICME) using the row data after subtraction of the blanks.

The current study was conducted to evaluate the effect of ICME as anti-Alzheimer plant material. Oxidative stress and increased cholinesterase activities are hallmarks for AD. Hence AChE activities, as well as the antioxidant activities of ICME, were evaluated. Firstly, ICME was found to have an effective anticholinesterase effect with an estimated IC50 of 24 ± 2.3 μg/ml. The concentration of 2 μg/ml showed a significant effect on the enzyme activities (Figure 1A). Also, DPPH scavenging activity assay showed that ICME possessed significant DPPH scavenging activity in 10 μg/ml concentration with an estimated EC50 of 103 ± 3.2 μg/ml (Figure 1B).

**Figure 2:**
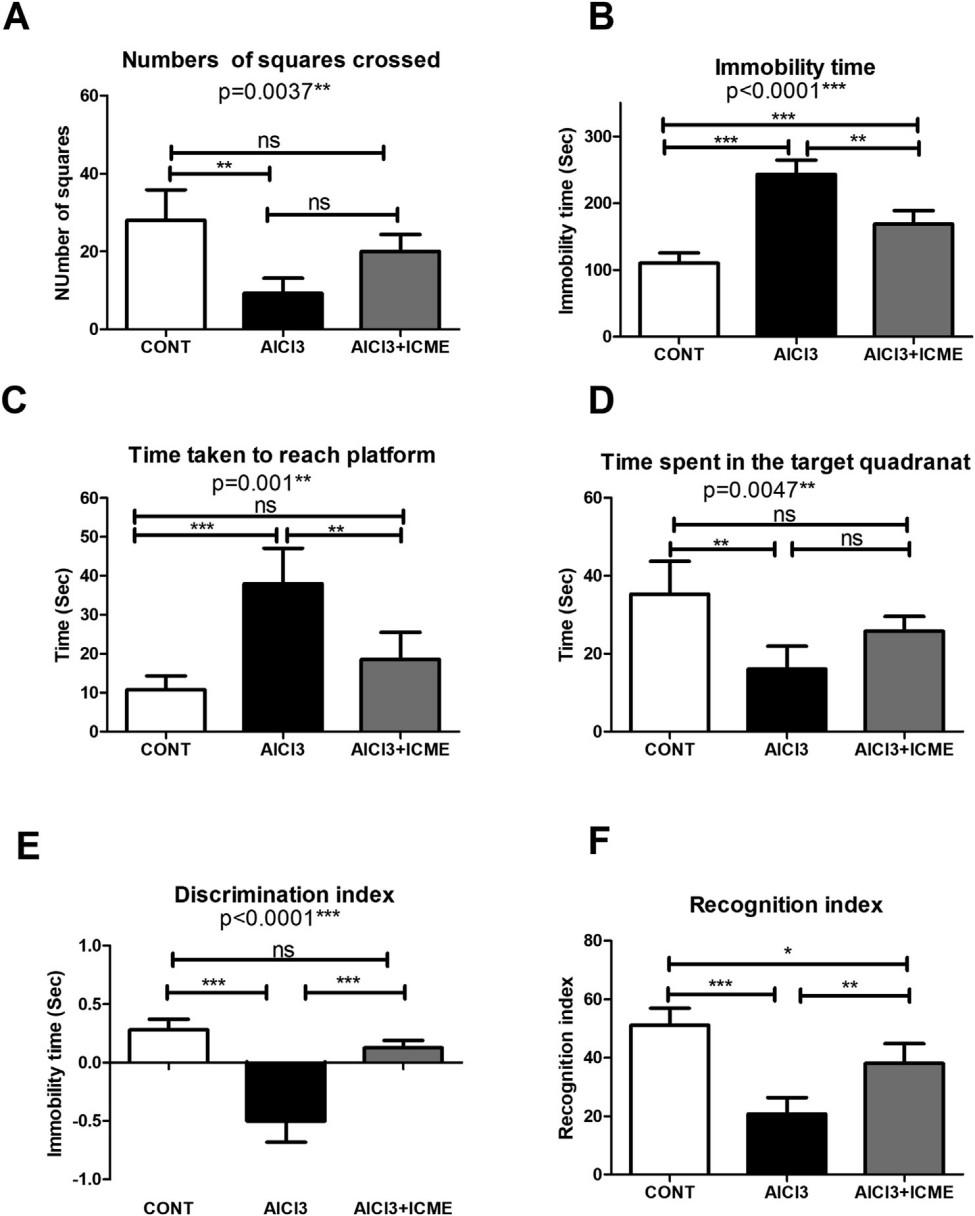
Neurobehavioral toxic effect of AlCl3 and the therapeutic and neuroprotective potentials of Indian Catechu methanolic extract (ICME) using the following assays parameters. 1. Open field test: A. squares explored, B. total immobility time; 2. Morris water maze: C.the time taken to escape to the platform, D. time spent in the target quadrant; 3. Novel object recognition test: E. discrimination index, 2. recognition index. Results were expressed as means ± SD. One-way ANOVA p-values were shown in the figure. ∗ means p-value <0.05, ∗∗ means p-value <0.01, while ∗∗∗ means p-value <0.001.

AlCL3 was used as a well-known toxicity model for AD. After the development of the model. Different behavioral and biochemical assays were conducted to evaluate the toxic effect of AlCl3 as well as the protective and therapeutic effects of ICME. Firstly, Three Behavioral studies were conducted. The open-field test showed that AlCl3 significantly decreased on the number of explored squares with a significant increase in a total time of immobility, while there was no effect observed on the rearing number and the number of fecal pellets (Figures 2A, B). Morris water maze test showed that AlCl3 significantly increased the time taken (s) by the rat from its immersion point till reaching the platform into the pool as well as the time spent by the rat in the target quadrant without the platform (Figure 2C, D). Novel object recognition assay showed that AlCl3 treated showed a significant decrease in both recognition and discrimination indices (Figure 2E, F). Interestingly, ICME showed a significant corrective effect on ALCl3 induced cognitive and behavioral dysfunction (Figure 2A-F).

**Figure 3:**
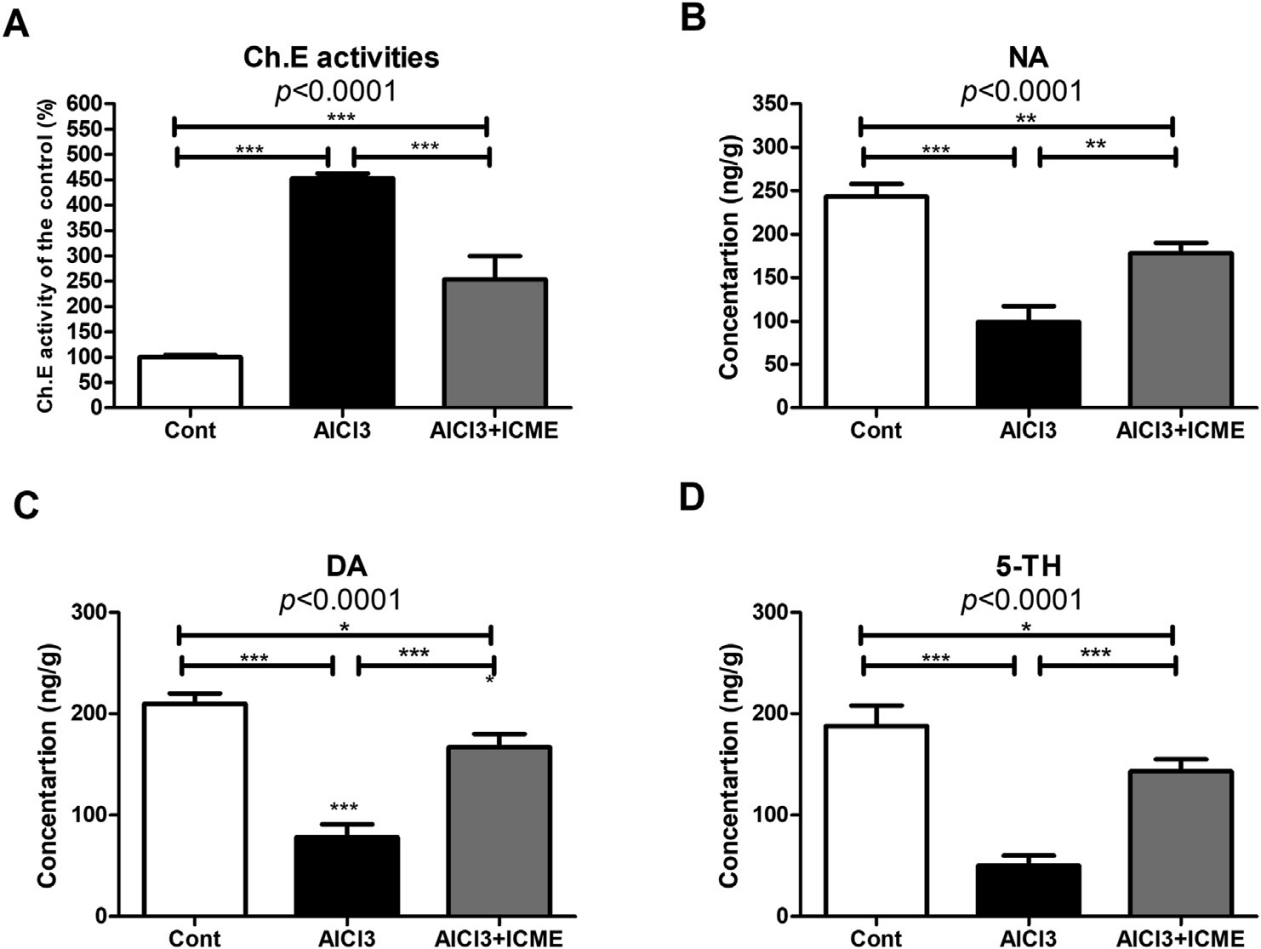
A shows the cholinesterase activity in the rats' brain tissue in response to AlCl_3_ neurotoxicity and the neuroprotective effect of Indian Catechu methanolic extract (ICME). B-D shows reversed-phase High Performance Liquid Chromatography (HPLC) with an electrochemical detector for the changes in the levels of biogenic amines [3B. Noradrenaline (NA), 4C. dopamine (DA), 3D. 5-hydroxytryptamine (5-HT)] in brain homogenates samples of the treated rats with AlCl3 alone or with both AlCl3 and ICME. Results were expressed as means ± SD. One-way ANOVA p-values were shown in the figure. ∗ means p-value <0.05, ∗∗ means p-value <0.01, while ∗∗∗ means p-value <0.001.

Regarding the treated animal cholinergic neuron activities. AlCl3 rats treated samples showed a significant increase in Ch.E activities in both cortical and hippocampal regions with more increase in the hippocampus samples. Group III brain samples showed less increase in Ch.E activities, which is significantly lower than the measured levels of the enzyme activities in AlCl3 alone treated rats (Figure 3A).

The effect of AlCl3 on the biogenic monoamines was evaluated. Reverse phase LC showed that AlCl3 caused a significant decrease in the biogenic amines NA, DA, and 5-TH by about 59.3 ± 7.4, 62.8 ± 6.2, and 73.4 ± 5.3 of the control levels, respectively. ICME showed treated groups showed that the biogenic amines NA, DA, and 5-TH were only reduced by about 26.7 ± 4.9, 20.5 ± 6.2, and 23.9 ± 6.4 of the control levels of the transmitters, respectively (Figure 3B, C, D).

**Figure 4:**
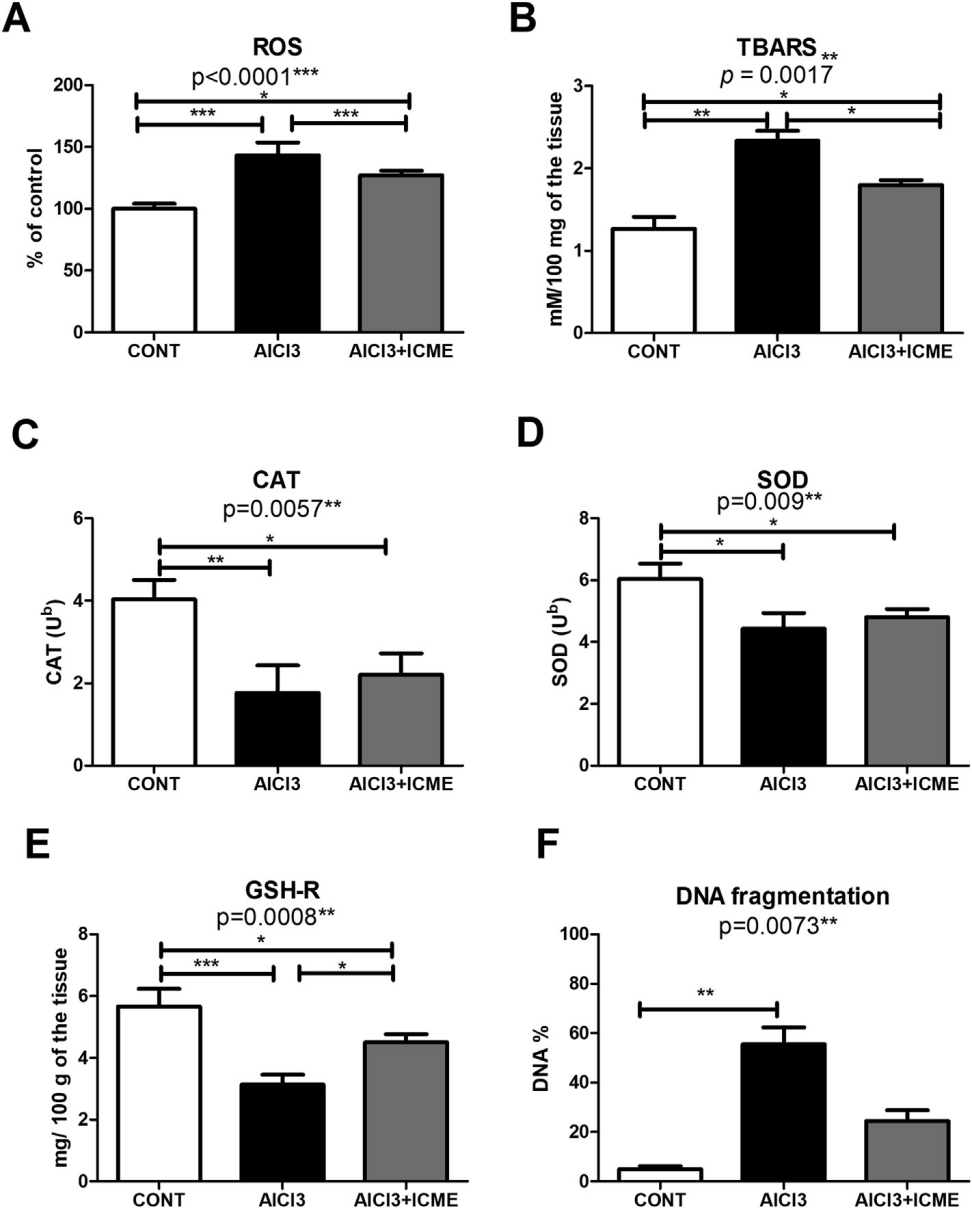
A-E shows the oxidative stress biomarkers in the treated rats' brains; A. reactive oxygen species (ROS), B. Lipid peroxidation product by thiobarbituric acid reactive substances (TBARS). C. Antioxidant enzyme catalase (CAT) activities, D. Antioxidant enzyme superoxide dismutase (SOD) activities. E. Reduced glutathione activities (GSH-R). F. DNA fragmentation assay data as a marker for AlCl3 induced genotoxic effect in the treated rats' brains. Results were expressed as means ± SD. One-way ANOVA p-values were shown in the figure. ∗ means p-value <0.05, ∗∗ means p-value <0.01, while ∗∗∗ means p-value <0.001.

Oxidative stress is known to play a role in AlCl3 neurotoxicity as well as the development of AD. Oxidative markers in the brain tissue of the rats’ were studied. Oxidative stress markers showed that AlCl3 caused a significant increase in ROS production in addition to a significant increase in lipid peroxidation with a parallel significant decrease in the activities of CTA and SOD and reduced glutathione. ICME was shown to significantly counteract the effect of AlCl3 effect of ROS, lipid peroxidation, and reduced glutathione without significant effect of the activities of CAT and SOD activities (Figure 4A-E).

Additionally, the genotoxic effect of AlCl3 on the treated Rats’ brains was studied using a DNA fragmentation assay. Data showed that AlCl3 significantly increased the percentage of fragmented DNA (~55%), while ICME treated rats showed only (~25%) fragmented DNA (Figure 4F).

**Figure 5:**
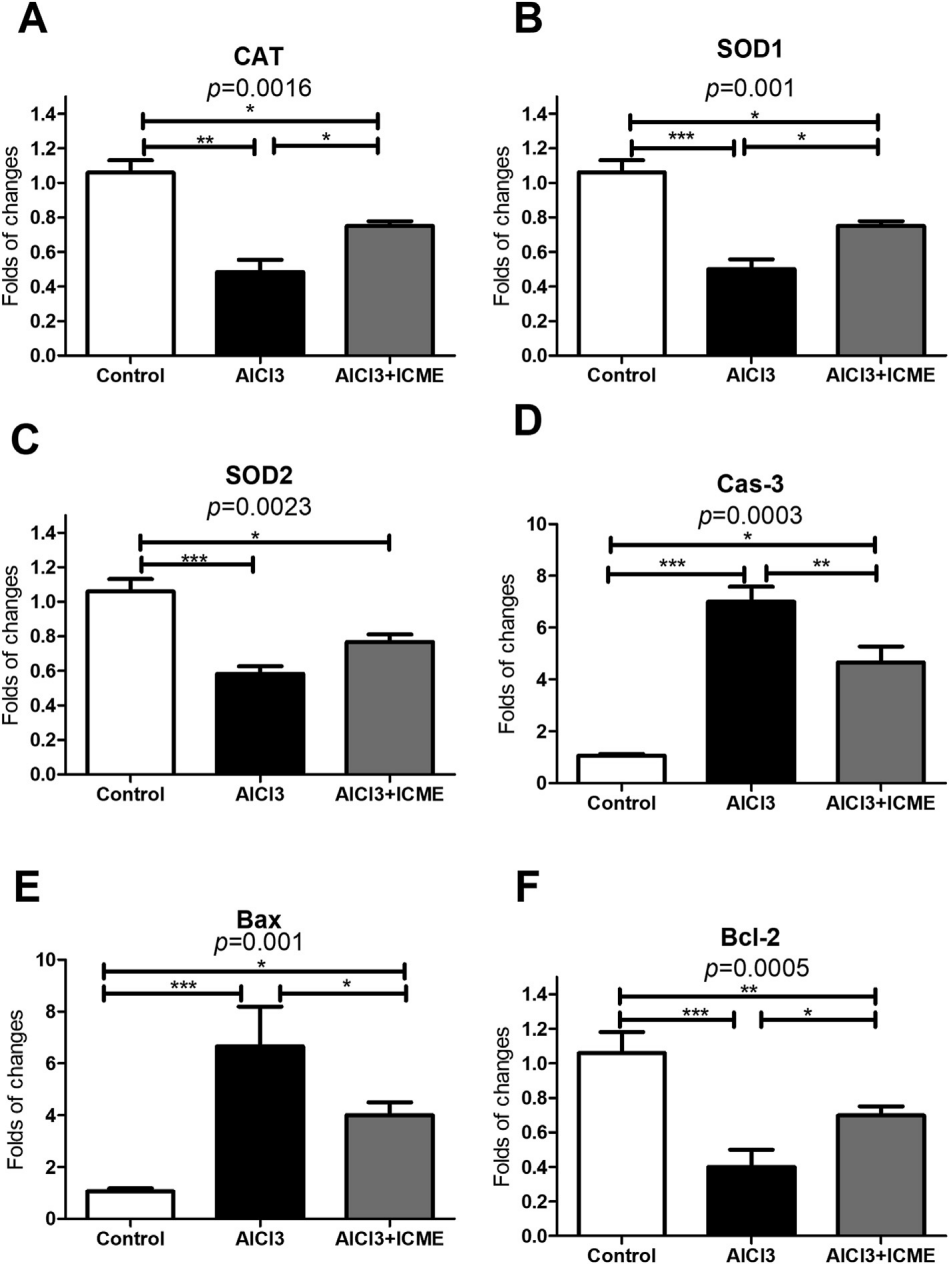
rt-PCR data regarding the effect of AlCl3 and Indian Catechu methanolic extract (ICME) on expression of the genes families involved in Oxidative stress and apoptosis: A. the expression of the gene coding for The antioxidant enzyme catalase (CAT) gene, B. The expression of antioxidant enzyme superoxide dismutase 1 (SOD1) gene. C. The expression of antioxidant enzyme superoxide dismutase 2 (SOD2) gene. D. The expression of caspase 3 (Cas-3) gene. E. The expression of pro-apoptotic (Bax) gene, F. The expression of anti-apoptotic (Bcl-2) gene. Data were sown as folds of changes in the studied genes expression relative to the control samples. Results were expressed as means ± SD. One-way ANOVA p-values were shown in the figure. ∗ means p-value <0.05, ∗∗ means p-value <0.01, while ∗∗∗ means p-value <0.001.

**Figure 6:**
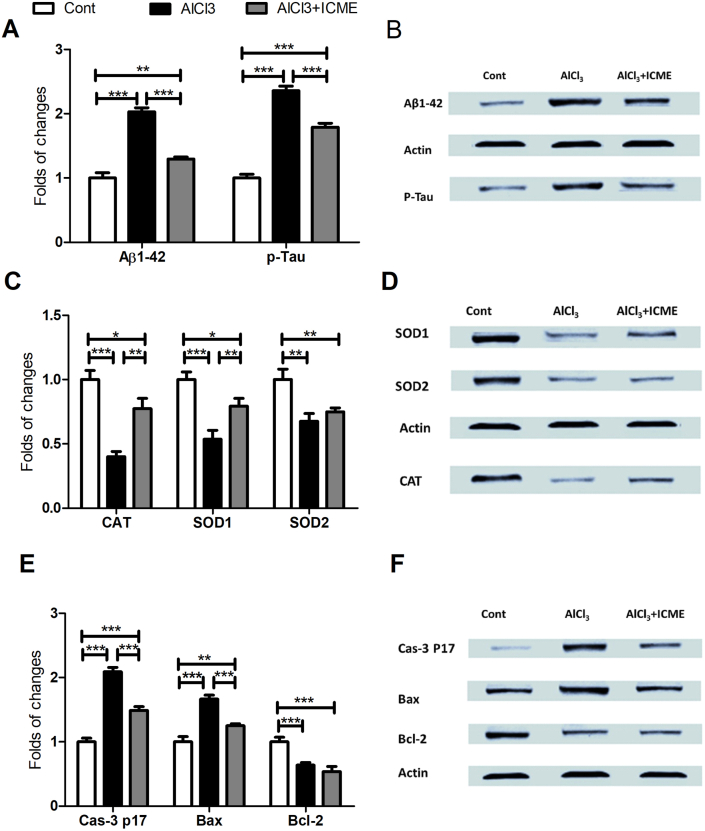
western blot data regarding the effect of AlCl3 and Indian Catechu methanolic extract (ICME) on protein levels of the beta amyloid 1–42 peptide (Aβ1-42) and phosphorylated Tau (P-Tau) proteins (hallmarks for Alzheimer disease) as well as other proteins involved in Oxidative stress and apoptosis: 5A and 5B show the western blot data for (Aβ1-42) and phosphorylated Tau (P-Tau). 5C and 5D show the protein levels of antioxidant enzymes catalase (CAT) gene, superoxide dismutase 1 (SOD1), and superoxide dismutase 2 (SOD2). 5E and 5F show the protein levels of caspase 3 (Cas-3), pro-apoptotic (Bax), The anti-apoptotic (Bcl-2) proteins. Data were sown as folds of changes in the studied proteins levels relative to the control samples. Results were expressed as means ± SD. One-way ANOVA p-values were shown in the figure. ∗ means p-value <0.05, ∗∗ means p-value <0.01, while ∗∗∗ means p-value <0.001.

The effect of AlCl3 in gene expression for gene families involved in apoptosis and oxidative stress was evaluated by rt-PCR. Data showed that AlCl3 caused a significant increase in expression of Caspase 3 and Bax proteins with parallel significant decrease in expression of genes Bcl2, SOD1, SOD2, and CAT. Also, ICME showed a significant beneficial effect to counteract the effect of AlCl3 in the treated samples brain tissues (Figure 5). Interestingly, western blot data were supporting to the data observed by rt-PCR (Figure 6).

**Figure 7:**
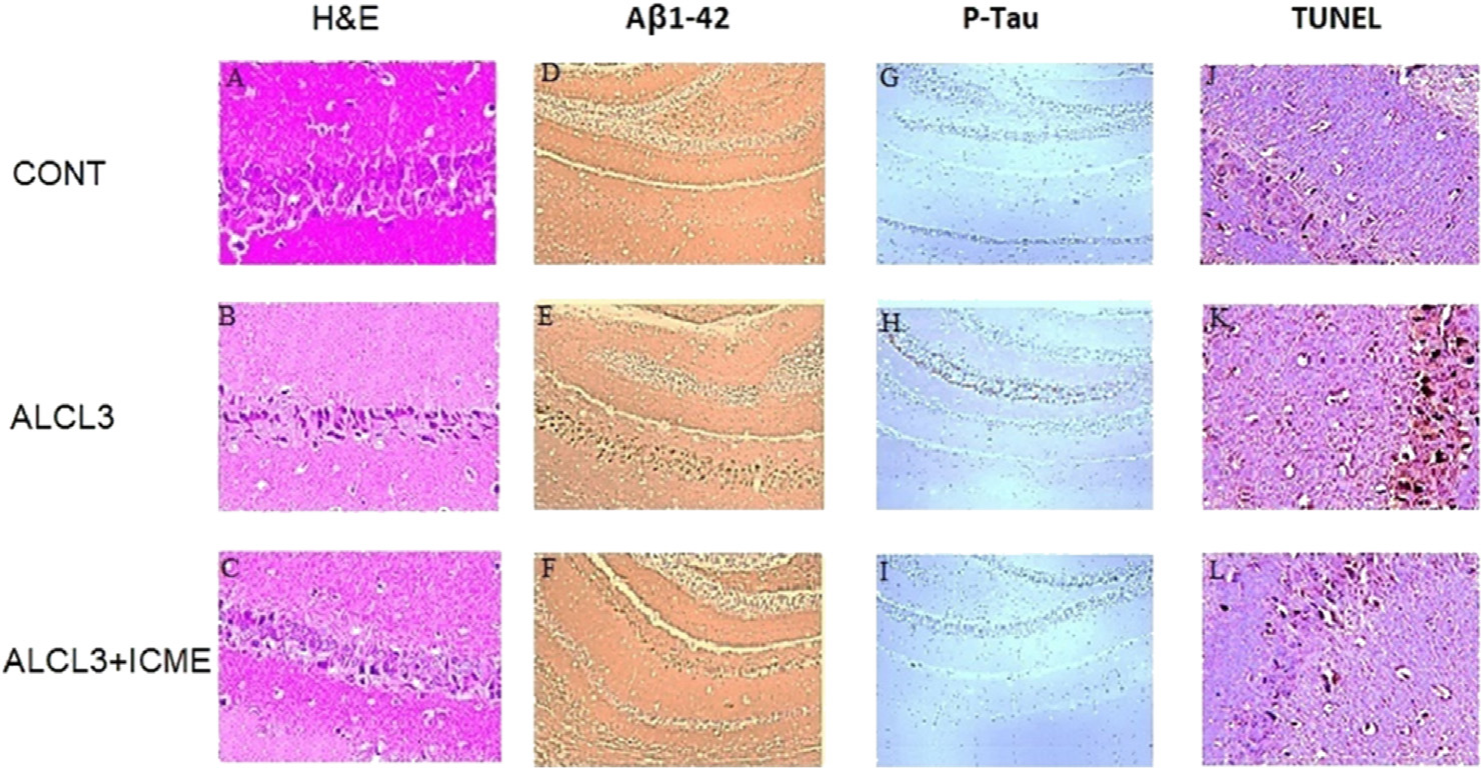
Morphological changes induced by AlCl3 and Indian Catechu methanolic extract (ICME) in rats' brain tissues. Brain tissue sections were stained by H&E (400) (A-C) and immune-stained for beta amyloid 1–42 peptide (Aβ1-42) (400) (D-F) and phosphorylated Tau (P-Tau) proteins (400) (G-I). Also brain tissue sections were stained by TUNEL (400) (J-L). Staining for Aβ1 42, P-Tau and TUNEL positive cells was measured by the image analyser.

**Figure 8:**
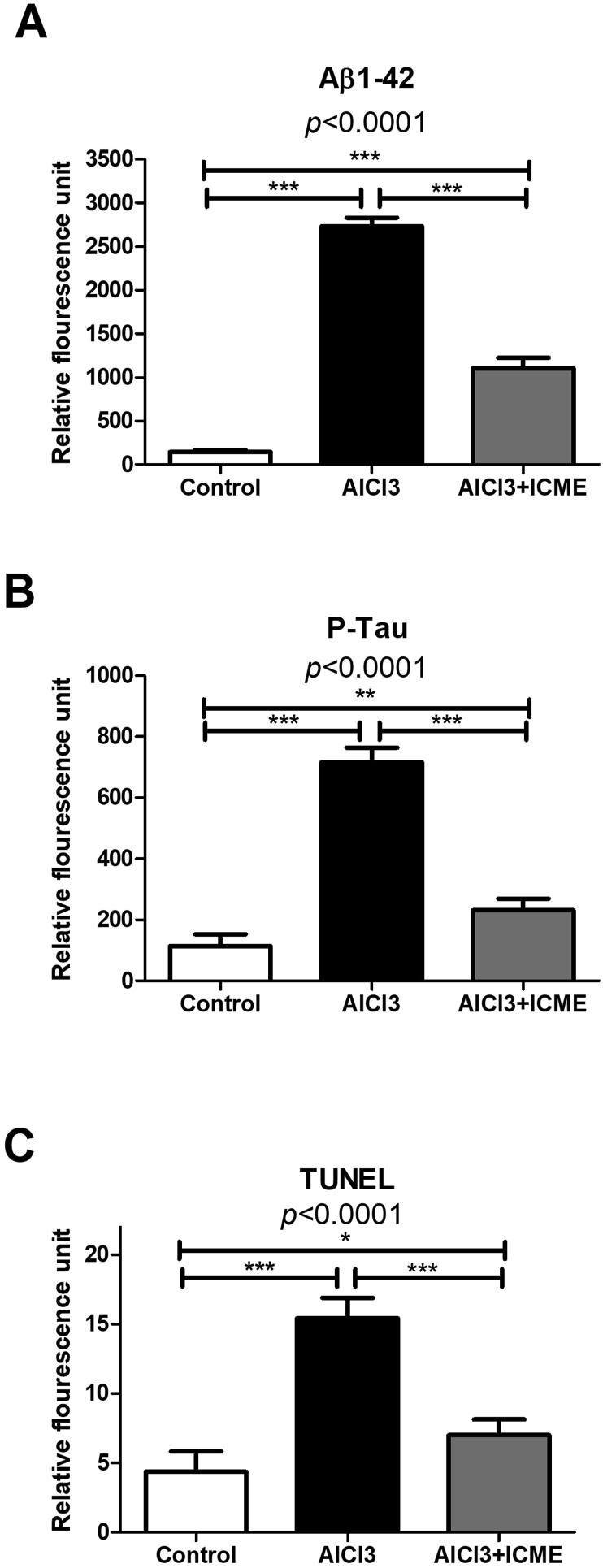
A and B show the quantitative immune-histochemistry data regading the effect of AlCl3 and Indian Catechu methanolic extract (ICME) on rats' brain tissues levels of beta amyloid 1–42 peptide (Aβ1-42) (A), phosphorylated Tau (P-Tau) proteins (B). While C shows TUNEL positive cells (C) levels in the untreated and treated animals. Results were expressed as means ± SD. One-way ANOVA p-values were shown in the figure. ∗ means p-value <0.05, ∗∗ means p-value <0.01, while ∗∗∗ means p-value <0.001.

Histopathological studies were done to evaluate the morphological changes induced by AlCl3 treated rats' hippocampuses as well as the protective and therapeutic role of the tested ICME. Hematoxylin and eosin data showed decreased neuron numbers in AlCl3 treated samples with pyknotic deeply stained nuclei and elongated axons in compassion to the control non-treated samples. ICME was found to significantly improved pathological changes which were found in the model rats' brains (Figure 7A-C). Immunostaining showed a significant increase in both Aβ1-42 and pTau in the model brains. ICME treated rats showed marked decrease in both proteins in rates treated with AlCl3 and ICME (Figures 7D-I and 8A, B). While TUNEL staining showed a significant increased apoptosis in AlCl3 model treated brains’ samples in comparison to controls, while the third group showed a significant therapeutic and protective effect of ICME (Figures 7J-I and 8C).

## 4 Discussion

The current study was conducted to evaluate the anti-Alzheimer effects of ICME using an AlCl_3_ toxic model of neurodegeneration that simulates many AD features (Ali et al., 2016; Prema et al., 2017; Singh et al., 2018). Aluminum has been shown to induce brain oxidative damage, neuronal death, cholinergic neuron degradation (Germano and Kinsella, 2005; Miu et al., 2006).

An ethnopharmacological approach of screening of the natural plant products against cholinesterase activities is a cost-effective and widely accepted strategy to develop new effective therapeutic agents. The present study showed that ICME can efficiently inhibit AChE in a dose-dependent fashion, which allow for further therapeutic potentials in AD as the other plant product which are marketed as medications for AD as Rivastigmine and galantamine with reported acetylcholinesterase inhibitors (Munoz-Torrero, 2008; Ballard et al., 2011; Syad and Devi 2014).

Besides, DPPH scavenging assay showed that ICME possessed a significant antioxidant effect. This antioxidant effect may be related to its contents of polyphenols and flavonoid compounds as shown previously (Patil and Modak, 2017) where the total phenolic content (gallic acid equivalent values) for 0.1%, 0.3%, 0.5% ICME was estimated to be 0.74 ± 0.068, 2.27 ± 0.071, 2.93 ± 0.041, respectively. And the total flavonoid content (equivalent of riboflavin) for 0.1%, 0.5%, and 1% ICME were found to be 2.30 ± 0.063, 3.41 ± 0.065, 5.58 ± 0.177 respectively. These phenolic and flavonoid compounds are known to have high antioxidant activities (Choudhary and Swarnkar, 2011). Hence ICME can play a role in many diseases in which oxidative stress is a major factor in its pathogenesis as the case in AD. This assumption is supported by other studies that showed the potential of some plants in AD based on their antioxidant and free radicle scavenging activities as Withanamides, *Bacopa monnieri* and *Centella asiatica* plants (Limpeanchob et al., 2008; Jayaprakasam et al., 2010; Roy et al., 2017).

We evaluated the beneficial effects of ICME –based on these anti-cholinesterase and antioxidants effects- against the AlCl3 induced model in rats. AlCl3 is known to induce behavioral, biochemical, and histopathological effects on the treated animals. The current animal model showed that AlCl3 toxic effect on the rats leads to significant behavioral alteration, in addition to a marked increase in cholinesterase activities in brain tissues accompanied by oxidative stress and genotoxicity. The cholinergic transmission has a high priority in the pathogenesis of AD as its dysfunctions are mainly related to the severity of dementia in AD patients (Amberla et al., 1993; Hampel et al., 2019). Aluminum is known to have a potent cholinotoxin with elevated Ch.E activities by modifying the secondary structure of the enzyme with marked impairment of the brain cholinergic transmission (Zatta et al., 2002; Kakkar and Kaur, 2011). On the other hand, ICME treatment led to a significant lowering in Ch.E activities in AlCl3 treated rats, which is following the invitro assays regarding its anticholinesterase effect of ICME. Interestingly, Behavioral tests showed that ICME significantly counteracted AlCl3-induced neurobehavioral changes related to cognitive abilities including memory and learning skills.

Regarding the effect of AlCl3 on the other biogenic monoamine transmission, the current study showed that AlCl3 significantly decreased brain tissue levels of noradrenaline, dopamine, and serotonin as observed previously (Liaqat et al., 2017; Haider et al., 2014). This mimics the brain levels of the monoamines among AD patients. Interestingly ICME was found to significantly alleviate the effect of AlCl3 on the biogenic monoamines. Decreased monoamines were suggested to have a prominent role in aging and development in neurodegenerative diseases (Palmer and DeKosky, 1993). Hence the currently reported effect of ICME to preserve the levels of monoamines in AlCl3 treated rats is also highly suggestive for their therapeutic effect against neurodegenerative disorders including AD. Interestingly, monoamines (noradrenaline, dopamine, and serotonin) modification was previously targeted as a therapeutic potential for *Moringa oleifera ethanolic extract which was reported to modify the monoamine in colchicine induced AD model in rats* (Ganguly and Guha, 2008).

Histopathological findings showed evident neurodegenerative effects in the form of, pyknosis and degeneration alongside excessive accumulation of beta amyloid and phosphorylated Tau proteins, which are considered hallmark pathologies of AD, as experienced in previous works using AlCl3(Ali et al., 2016; Prema et al., 2017; Singh et al., 2018). Deposition of extracellular beta amyloid and intracellular phosphorylated Tau is known to play a significant role in the pathogenesis of AD. Both AlCl3 induced inflammation and oxidative stress were suggested to play a significant role in the deposition of both proteins (Perry et al., 2002; Tanzi et al., 2004; Xiong et al., 2013; Ma et al., 2017; Sahu et al., 2014). In addition, beta amyloid was found to induce oxidative stress, which plays a significant role in the progression of AD (Butterfield et al., 2013). ICME significantly decreased both protein depositions in AlCl3 treated rats’ brain tissues. This may be due to its significant antioxidant effects shown in the study and its protective effect on the antioxidant enzymes CAT and SOD. In addition, previous studies had reported the anti-inflammatory effect of the plant extracts as Chandra et al. (2019). The anti-amyloid effect of some plants was previously evaluated as a therapeutic modality for AD as *Ginkgo biloba* which showed an anti-amyloid aggregation effect. Based on this effect, further studies on *Ginkgo biloba* showed that daily intake of 240 mg of *Ginkgo biloba* can decrease the incidence of AD (DeKosky et al., 2008).

## 5 Conclusion

As altered cholinergic neurotransmission and oxidative stress play important role in the pathogenesis of AD, the current findings can support the use of ICME as a potential therapeutic for AD as it shows marked anticholinesterase effect and significant antioxidant effect. Rats’ study showed that ICME significantly improved the cholinergic neurotransmission and reduced the genotoxic effect of AlCl3 with much more improvement in the histopathology and biochemical findings.

## Declarations

### Author contribution statement

Ekramy Elmorsy; Eman Elsharkawy; Fahad A. Alhumaydhi: Conceived and designed the experiments; Performed the experiments; Analyzed and interpreted the data; Contributed reagents, materials, analysis tools or data; Wrote the paper.

Mohamed Salama: Conceived and designed the experiments; Wrote the paper.

### Funding statement

This research did not receive any specific grant from funding agencies in the public, commercial, or not-for-profit sectors.

### Data availability statement

Data included in article/supplementary material/referenced in article.

### Declaration of interests statement

The authors declare no conflict of interest.

### Additional information

No additional information is available for this paper.
